# Review of upper extremity passive joint impedance identification in people with Duchenne Muscular Dystrophy

**DOI:** 10.1186/s12984-024-01537-0

**Published:** 2025-01-25

**Authors:** Suzanne J. Filius, Kyriacos Papa, Jaap Harlaar

**Affiliations:** 1 Department of BioMechanical Engineering, Delft University of Technology, Mekelweg 2, Delft, 2628 CD South-Holland The Netherlands; 2 Department of Orthopedics and Sports Medicine, Erasmus MC University Medical Center, Dr. Molewaterplein 40, Rotterdam, 3015 GD South-Holland The Netherlands

**Keywords:** DMD, Upper extremity, Joint stiffness, Passive joint impedance, Muscular Dystrophy, Neuromuscular disorders

## Abstract

Duchenne Muscular Dystrophy (DMD) progressively leads to loss of limb function due to muscle weakness. The incurable nature of the disease shifts the focus to improving quality of life, including assistive supports to improve arm function. Over time, the passive joint impedance (Jimp) of people with DMD increases. Force-based controlled motorised arm supports require a clear distinction between the user’s movement intention and passive forces, such as passive Jimp. Therefore, Jimp identification is essential. This review aims to define Jimp, identify factors influencing it, and outline experimental methods used for quantification, with a focus on the upper extremities in DMD. A literature review was performed in May 2021 and updated in March 2024 using SCOPUS, PubMed, IEEEXplore, and WebOfScience. The results reveal confusion in definitions and show various Jimp measuring practices for both DMD and individuals without muscle weakness. This study presents an overview and lists important parameters affecting passive Jimp, such as the joint’s position, velocity and the multi-articular nature of the upper arm muscles. For personalised passive Jimp compensation in arm supports, ramp-type perturbations with constant velocity across the full joint range appear most optimal for identifying the elevated and non-linear nature of the passive Jimp in DMD.

## Introduction

Duchenne Muscular Dystrophy (DMD) is an inherited disease resulting from the mutation of the X-chromosome dystrophin gene, leading to the absence of the structural protein dystrophin [1–3]. It is the most prevalent muscular dystrophy, mainly affecting boys, with an incidence rate of about 1:5000 live male births [4, 5]. Patients cope with loss of ambulation, loss of upper extremity (UE) function, scoliosis, and respiratory and cardiovascular complications, with the latter two eventually leading to death [1–3, 6, 7]. The lack of dystrophin causes various muscle morphological changes, with muscle fibres losing their ability to repair after several deterioration-regeneration cycles, leading to permanent degradation and replacing them with adipose (i.e., fat) and connective tissue (i.e., fibrosis) [1, 3, 8, 9]. This process causes muscle loss [1] and the formation of joint contractures [2]. Further weakening of the muscles, combined with joint contractures, limits the joint’s range of motion (ROM) [1, 9, 10]. This results in arm-function deterioration, hindering the activities of daily living and social participation [10–12], making them more dependent on family and caretakers [2].

Arm-assistive devices can improve the quality of life of people with DMD by compensating for the arm’s weight and assisting the arm functionality [2, 13]. An example of such a device is the passive Wilmington Robotic Exoskeleton (JAECO Orthopedic, Hot Springs, AR). Unfortunately, as the disease progresses, even with passive assistance, muscle strength becomes insufficient, making boys with DMD unable to overcome the friction and inertia of the passive supports, and passive forces exerted on their arms [2, 13, 14]. This makes it difficult to raise their arms above their heads, lift objects with additional weight [13], and perform downward movements [15].

Due to muscle weakness and morphological changes in the muscles, the so-called passive joint impedance (Jimp) increases, making movement even more difficult. Jimp refers to the resistance against a movement, often referred to as joint stiffness in the clinical field. As a consequence, the functional ability decreases even further. Even with arm supports that compensate for the arm’s weight, the functional ability can be limited by this passive Jimp [14] and compensating for the passive Jimp in assistive arm supports seems promising.

Straathof et al. [6] showed in their study with people with DMD that providing Jimp compensation with the planar active A-Arm support system (Flextension Project, The Netherlands) increased the functional ROM of the users’ arm. Moreover, Lobo-Prat et al. [2, 14] showed with the UR5 Robotic arm (Universal Robots, Denmark) that compensating for both arm weight and passive Jimp leads to an increased horizontal and vertical workspace of the arm in an individual with DMD, compared to weight compensation alone.

Unlike passive, active-assistance devices can compensate not only for the weight of the arm but also for its passive Jimp, including stiffness, damping, and inertia forces [2, 6, 14]. While different control methods of these devices exist, force-based control requires a clear distinction between the user’s voluntary and passive forces (weight and passive Jimp) [2]. Thus, the passive Jimp and the gravity component must be correctly estimated to properly identify the user’s intention and improve the device’s control [2, 6, 13, 16].

Unfortunately, the literature lacks sufficient quantitative data on the levels and behaviour of passive Jimp in individuals with DMD, limiting the development of force-based compensation models in assistive technologies [17]. Moreover, specialists in various fields [13, 14, 18–20] use different terms and methods to describe and quantify passive Jimp. Therefore, there is a need to clarify the definitions used for passive Jimp and the parameters that affect it. For this reason, the study provides (1) an insight into what passive Jimp is, (2) parameters affecting Jimp, and (3) an overview of the experimental methods in the literature measuring the Jimp of the upper extremities in people with DMD and without muscle weakness.

## Methods

**Fig. 1:**
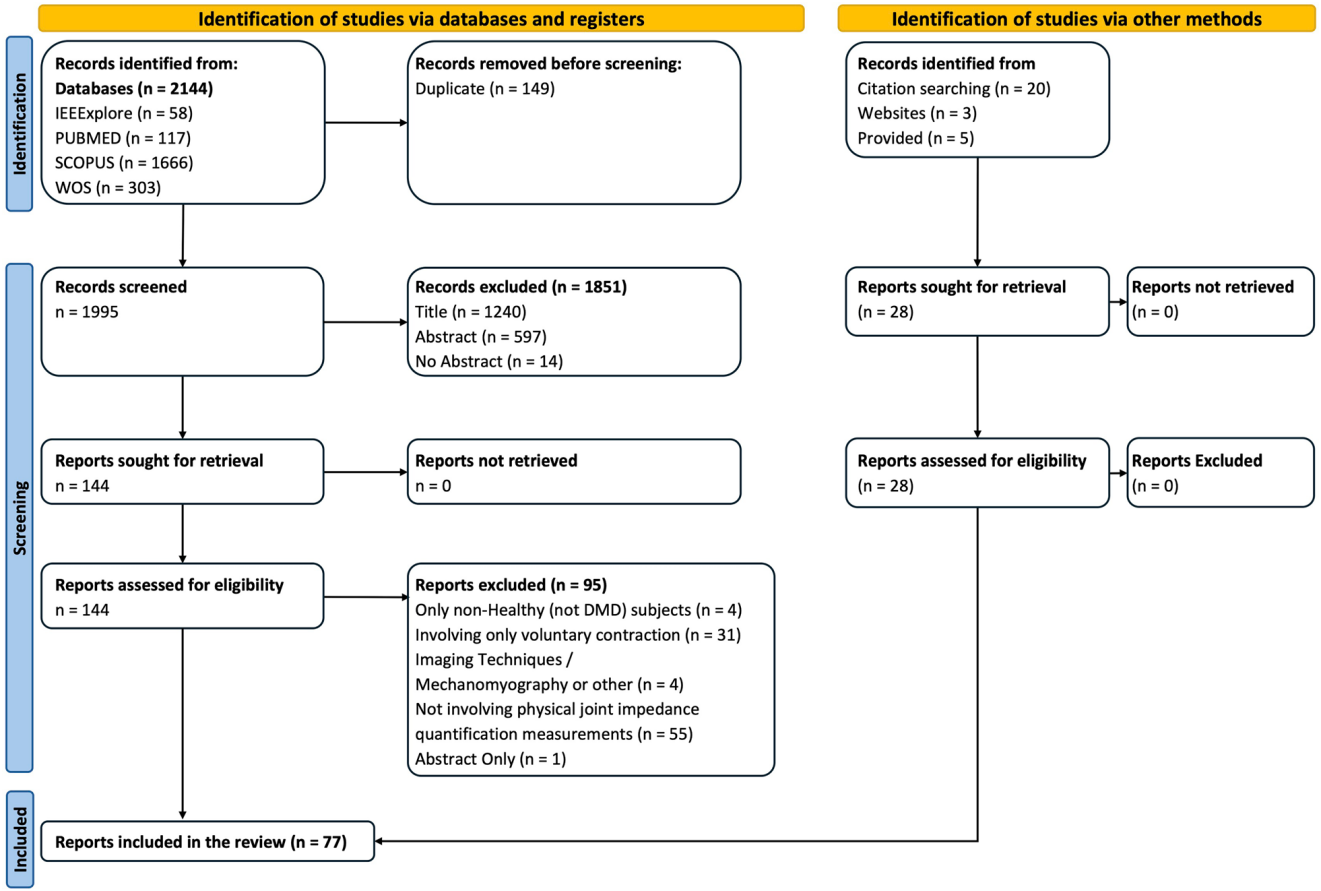
Prisma Flowchart process for the selection of the results of the review [21]

The Prisma flowchart in Fig. 1 displays an overview of the process.

### Literature search

In May 2021, a single reviewer (KP) conducted a literature study in four electronic databases: SCOPUS, PubMed, IEEEXplore, and WebOfScience. This search was updated in March 2024. The following keywords and Medical Subject Headings (MeSH) Terms were used in the search: *stiffness, co-activation, contractures, ROM, elasticity, excursion, and fibrosis*, focused only on the muscles and joints (*shoulder, elbow, wrist*) of the *upper extremities and limbs*. Finally, the terms *Duchenne, Becker, muscular dystrophy/ies and neuromuscular diseases* were used. The terms were refined by reviewing multiple results to ensure relevance and were explicitly adjusted for each database. Where applicable, they were combined with additional filters. In all databases, only studies involving adolescents and adults were included. Where possible, non-human results were excluded. Detailed database-specific search strings are available in Appendix I: Search Terms Next, all the results were imported into EndNote X9.3.3 software (Clarivate) and cleared from duplicates.

### Screening

Based on the title and abstract, results written in a non-English language, contained no abstract, and papers that concern DMD but describe treatments with toxin or surgery, gene, or protein mutations were excluded. Moreover, papers that only used imaging techniques to measure the stiffness or rigidity of the muscles were excluded. Additionally, papers describing assistive arm supports for people with DMD, that do not consider passive Jimp identification, modelling or compensation were excluded.

Since the number of results related to passive Jimp identification and DMD was very small (5), also papers describing experiments to identify passive Jimp in non-disabled participants were included. However, papers focused solely on sports and athletes or those only involving experiments requiring a form of voluntary muscle contraction during identification were excluded. So, studies quantifying Jimp in people with DMD (with or without voluntary contraction) and non-disabled participants (including only those describing experiments without voluntary contraction) were included. Moreover, studies providing general information about relevant definitions, and parameters affecting the Jimp were included.

Finally, a full review of the selected papers was carried out. Any cross-references to studies about experiments and Jimp attributes were added to the pool for further examination.

## Results

### Definition of passive joint impedance

Various terms like stiffness, impedance, elastic coefficient, tonus, hypertonia, spasticity, and rigidity were identified in the (clinical) literature, with professionals having difficulty distinguishing some of them [19, 20]. We quoted the definitions in Table 1 for clarity. To illustrate, Roberson and Giurintano [22] define ‘*joint stiffness*’ as “a limitation in the ROM of a joint or a resistance encountered while the joint is moved through its ROM”. While Boon et al.[23] defined ‘*mechanical impedance*’ as “the mechanical resistance that is exerted in response to passive motion”, which is also referred to as ‘*rigidity*’ in the medical terminology [23]. Moreover, ‘*muscle tone*’ characterises the resistance to an externally forced movement [24]. Wiegner and Watts [25] refer to ‘*tone*’ and ‘*stiffness*’ as the same thing, similar to Malhotra et al. [26] who describe tone as stiffness [27]. Chuang et al. [28] mention poor discrimination between increased muscle tone and soft-tissue stiffness. Similar to ‘*spasticity*’ [27] and ‘*rigidity*’ [29], muscle tone is influenced by the innervation of the muscles [24, 28]. A clear definition for tonus and spasticity is provided in Table 1.

Maggioni et al. [30] give a clear definition of joint impedance and distinguish it from the clinically often used term joint stiffness. They describe ‘*joint impedance*’ as the “force generated by changes in position (e.g., stiffness, non-elastic forces), in velocity (e.g., viscosity, damping) and in acceleration (e.g., inertia)” [30, 31]. Therefore, the term joint impedance is, in this case, better than joint stiffness since it consists of more components than pure stiffness. Joint stiffness alone can only describe the static property of a joint, while joint viscosity and limb inertia are needed to characterise its dynamic resistance against an external perturbation [32], where perturbation refers to an externally applied force or motion that alters the state of the limb.

Moreover, they clearly distinguish the active and passive forms of Jimp. *Passive* Jimp results from the passive biomechanical properties of the muscles, tendons, and tissues around the joint and limb inertia [30]. In contrast, the *active* form is a result of the response to reflexes or resistance produced by (non-reflexive) muscle contractions, such as tone and spasticity, which both are absent in DMD [1]. Therefore, *passive* Jimp is the primary focus of this paper.

**Table 1:** Comparison of descriptions

Term	Descriptions
Joint stiffness	“A limitation in the ROM of a joint or a resistance encountered while the joint is moved through its ROM” [22].
Mechanical Impedance	“The mechanical resistance that is exerted in response to passive motion” [23].
	“Mechanical impedance is the force resistance to perturbations of state” [33].
Mechanical admittance	Denotes the deformation change in response to a load disturbance. The inverse of mechanical impedance [34].
Tonus	“The state of activity or tension of a muscle beyond that related to its physical properties, that is, its active resistance to stretch. In skeletal muscle, tonus is dependent upon efferent innervation” [35].
Myotonia	“Prolonged failure of muscle relaxation after contraction. This may occur after voluntary contractions, muscle percussion, or electrical stimulation of the muscle. Myotonia is a characteristic feature of myotonic disorders” [36].
Rigidity	“Continuous involuntary sustained muscle contraction which is often a manifestation of basal ganglia diseases. When an affected muscle is passively stretched, the degree of resistance remains constant regardless of the rate at which the muscle is stretched. This feature helps to distinguish rigidity from muscle spasticity” [37].
Spasticity	“A motor disorder characterized by a velocity dependent increase in tonic stretch reflexes (muscle tone) with exaggerated tendon jerks, resulting from hyper excitability of the stretch reflex, as one component of the upper motor neurone syndrome” [38].

### Experimental methods to quantify the joint impedance of the upper extremity

This literature review yielded 47 studies looking into the mechanical properties of the UE, dating from 1973 to 2022. Table 2 summarises the identified experimental studies. The studies varied in the imposed movements, the joints of the upper extremity in focus, and the study population.

**Table 2:** Studies investigating the joint impedance of the upper extremities

Perturbation^a^	ROM	Joint(s)	Subjects	Device/ Examiner	Experiment Description	Source
**Duchenne Muscular Dystrophy Subjects**						
Ramp	Full horizontal arm’s workspace (2D planar)	Full Arm	3 DMD (Brooke 4–6) (18–23 yr)	D	Measured the passive forces of the relaxed arm by passively moving it in the horizontal plane.	[2]
Ramp	Full joint workspace (1D)	Elbow	3 DMD (Brooke 5) (21–22 yr)	D	Measured elbow compensation (gravity and joint stiffness) forces with a constant velocity while the arm is relaxed.	[16]
Ramp	Full horizontal arm’s workspace (2D planar) $\approx {32} \hbox { cm}\times 17 \hbox { cm }={544 \hbox { cm}}^{2}$	Full Arm	1 DMD (Brooke 5) (24 yr)	D	Low-velocity passive arm movement creates a 2D force field in the transverse plane.	[6]
Ramp	Full horizontal arm’s workspace (2D planar)	Full Arm	1 DMD (23 yr)	D	Passive arm forces by sweeping the arm in the horizontal plane in front of the subject’s workspace.	[14]
(1) QR, (2) FP	(1) Not Specified, (2) $3^{\circ }$ peak-to-peak harmonic angular displacement	Elbow	22 DMD boys ($13.55\pm 3.03$ yr, 9–21 yr), 15 healthy boys ($11.02\pm 1.66$ yr, 9–15 yr)	D	Performed isometric Maximum Voluntary Contraction (MVC) tests, Quick-Release (QR) and sinusoidal perturbations (SP) tests with sub-maximal MVCs.	[9]
**Healthy Subjects**						
Static	Nine hand positions, 10 cm apart forming a $3\times 3$ grid (shoulder ${12}^{\circ } - {70^{\circ }}$, elbow ${48}^{\circ } - {110}^{{\circ }}$)	Full Arm	9 (22-41yr) (8 Right-Handed (RH) males)	D	While the subject was relaxed, they estimated arm stiffness ellipses by obtaining measurements immediately before applying perturbations to the hand.	[43]
Ramp-and-Hold	7 mm	Shoulder, Elbow	11 “young” (4 males) ($27\pm 5$ yr) and 11 “old” (7 males) ($58\pm 12$ yr)	D	(1) Baseline session measuring the passive arm impedance and (2) Perturbation sessions (clockwise and isotropic) with subjects resisting force perturbations in eight different directions (${0^{{\circ }}}$, $\pm {45^{{\circ }}}$, $\pm {90^{{\circ }}}$, $\pm {135^{{\circ }}}$ and ${180^{{\circ }}}$).	[50]
PRBS	$\pm {2.5^{{\circ }}}~(\pm {0.045} \,{{\textrm{rad}}})$ in IER and $\pm {1.5^{{\circ }}}~(\pm {0.025} \,{\textrm{rad}})$ in horizontal ABD/ADD	Shoulder	15 males and females ($29\pm 5$ yr)	D	Rapid perturbations in different directions (IER, ABD and ADD) while subjects maintained different MVC torque levels of 0% (passive) to 40%, at 10% increments.	[45]
Ramp	Not Specified	Shoulder	20 males ($37\pm 7.47$ yr)	D	Involved inferior-directed translation of the glenohumeral joint where they gradually applied to the humeral head a preload of 10 N followed by a target load of 80 N.	[55]
Step	Max $\approx {150}^{\circ } \hbox { to } {195^{\circ }}$	Shoulder	10 males (24–29 yr) ($25.9\pm 1.79$ yr)	E	Examiner applied a tensile load of 30 N to the humerus until achieving a 4 N m IER. Tested for humerus elevation $({30^{\circ },\,45^{\circ },\,60^{\circ },\,90^{\circ },\,120^{\circ } \hbox { and } 135^{\circ }})$, and planes anterior and posterior to the scapula (${30^{\circ } \hbox { and } 60^{\circ }}$).	[84]
Static	Not Specified	Shoulder, Elbow	18 females ($19.8\pm 1.3$ yr)	D	Measured the mean passive shoulder joint extension moment before stimulation at different shoulder and elbow positions.	[41]
FP	Small Amplitude	Shoulder	7 males ($33\pm 7.9$ yr)	D	Small-amplitude abduction perturbations to the shoulder while the user is relaxed or minimising the background torque error with sub-maximal MVCs.	[32]
Ramp-and-Hold	${10^{\circ }}$	Elbow	10 RH (5 males) ($24.4\pm 2.7$ yr)	D	Examining elbow joint movement at different muscle contraction levels and velocities.	[48]
Repeating Ramp	${60^{\circ } \hbox { and } 120^{\circ }}$	Elbow	9 ($24.4\pm 4.2$yr)	E	Measured neural and non-neural torques, position, and velocity while the examiner moved the subject’s elbow joint passively.	[85]
PRBS	0.03 rad	Elbow	15 ($53.5\pm 9.6$yr)	D	Applied pseudo-random perturbations to the elbow joint to measure the intrinsic and reflex dynamic stiffness.	[86]
FP	Amplitude standard deviation $\sigma ={1.5^{{\circ }}}$	Elbow	5 (25–40 yr)	D	Applying standard perturbations to the elbow joint to evaluate intrinsic and reflex properties before and after fatigue with 0–50% MVCs.	[44]
(1) Torque Pulses, (2) Ramp	(1) 1 to 7 N m (2) $\approx {60^{\circ }}$ ($4\times$ EXT and then $4\times$ FLX sequences)	Elbow	19 (11 males) (20–78 yr, median 32 yr)	D	Conducted preliminary and formal experiments to examine limb velocity and muscle reflex contraction of the relaxed joint. Used torque pulses of varying levels and different levels of torque for elbow FE movements.	[25]
(1) FP, (2) STD, (3) Torque Pulse	(1) 0.005 to 0.5 rad, (2) Varying amount $0\pm {0.5}\, {{\textrm{rad}}}$, (3) Superimposed for pulse amplitudes up to 0.05 rad	Elbow	5 (3 males) (21–37 yr)	D	Investigated (1) the Frequency Response of the elbow joint over different amplitudes and frequencies, applied (2) Static torque displacements and (3) Test Pulse simulations over different joint positions.	[47]
(1) Step, (2) Ramp	(1) Not Specified, (2) ${2^{\circ }}$	Elbow	1 male adult	D	Examine the passive and reflex-mediated muscle stiffness of the relaxed elbow through (1) step (different amplitudes) and (2) ramp (different velocities) tests, with different isometric contraction levels preceding the perturbation onset.	[87]
Repeating Ramp	(1) ${0.06}\, {{\textrm{rad}}}$ and (2) $\le {0.4} \, {\textrm{rad}}$ at elbow ${90^{{\circ }}}$	Elbow	5 (4 males) (20-26yr)	D	Measured the passive joint impedance of the elbow for different velocities over the same amplitude and vice versa.	[23]
Ramp	${18^{{\circ }}}$ in 2D FE-RUD space	Wrist	13 (7 RH males) (19–55 yr), 3 LH males (23–60 yr)	D	Different target positions of passive wrist FE and Radio-Ulnar Deviation (RUD) movements.	[57]
Ramp-and-Hold	0.15 rad peak-to-peak	Wrist	8 (5 males), ($33\pm 9$ yr)	D	Wrist dynamic model behaviour parameters are estimated through ramp-and-hold perturbations of different torque levels and velocities. Subjects were both relaxed and applying torque against the manipulator.	[51]
Random Torque Perturbations	Mean displacement amplitude across subjects: (FE, RUD and PS) = $(5.4^{\circ }, 5.1^{\circ } \hbox { and } 4.1^{\circ })$	Forearm and Wrist	8 RH (6 males) ($27.1\pm 3.4$ yr)	D	Applied random force perturbations in three directions to investigate wrist FE, RUD, and forearm pronation/supination (PS).	[62]
PRBS	${0} \pm 10^{\circ }$	Wrist	14 RH (3 males) ($27\pm 2.9$ yr)	D	Used small amplitude, high-velocity (${100^{{\circ }}/\textrm{s}}$) wrist displacements to evaluate wrist biomechanical properties ($\mathrm {S_{MECH}}$) and wrist position sense before and after a fatigue task.	[73]
Torque Pulse	Peak wrist FLX (${33.2} \pm {9.1^{{\circ }}}$) and EXT (${32.8} \pm {5.8^{{\circ }}}$) angles	Wrist	10 RH males ($22.7\pm 2.7$ yr)	D	Wrist-joint rotational stiffness estimation through perturbations with three sub-maximal hand-grip MVCs. Examined the effect of co-contraction and perturbation anticipation.	[79]
Ramp	Sphere radius ${15^{{\circ }}}$ in PS-FE-RUD space	Wrist	10 RH (5 males) ($24\pm 5.42$ yr)	D	Passive Wrist FE at different forearm PS and RUD positions.	[54]
Repeating Ramp	${37^{{\circ }}}$ FLX to ${36^{{\circ }}}$ EXT and from ${16^{{\circ }}}$ RD to ${28^{{\circ }}}$ UD	Wrist	15 RH (7 males) (20–27 yr)	D	Examined wrist stiffness from its neutral position to 24 peripheral targets around the FE/RUD workspace of the wrist joint.	[72]
Ramp-and-Hold	$2^{\circ }, 4^{\circ } \hbox { and } 8^{\circ }$	Wrist	7 (3 males), ($38\pm 12$ yr)	D	Assessed wrist joint properties with ramp-and-hold perturbations at different amplitudes and FLX torque combinations.	[46]
Ramp-and-Hold	(1) FE$=$ −0.8 to 0.6 rad, RUD$=$ −0.3 to 0.3 rad, PS$=$ −0.8 to 0.8 rad, (2) 0.3 rad	Wrist	10 RH (7 males), mean age 34 yr, 34–42 yr	D	Conducted (1) 1D (FE, RUD, PS) and (2) 2D (FE-RUD space) passive wrist movements with a constant velocity ($< {0.2}\, {{\textrm{rad}}}$).	[68]
Ramp-and-Hold	Not Specified	Wrist	8 (4 males) ($25\pm 4$ yr)	D	Applied random Ramp-and-Hold wrist extension perturbations of different combinations of angular velocities and target torques to examine the dependency of the SRS elastic limit on joint velocity.	[49]
Static	${10^{{\circ }}}$ increments of MCP joint at ${60^{\circ }, 0^{\circ } \hbox { and }-60^{\circ }}$ wrist FE angles	Wrist, Fingers (MCP)	6 (3 males) (25-28yr)	D	Examined metacarpophalangeal (MCP) joint stiffness for three different wrist positions with incremental fingertip force measurement.	[40]
Static	Maximum FE limits of the MCP joint in ${10^{{\circ }}}$ increments for wrist FE angles: ${60^{\circ }, 0^{\circ } \hbox { and }-60^{{\circ }}}$	Wrist, Fingers	4 (2 males) (25-28yr)	D	Measuring total passive torque at different wrist and fingertip positions.	[39]
Repeating Ramp	${50 ^{{\circ }}}$	Wrist	48 (24 males) (21–70 yr, mean age $45.3\pm 13.7$)	D	Measured the Intrinsic Stiffness Index, Total Stiffness Index and Stretch Reflex Threshold Speed through different velocity wrist extension ramp tests.	[24]
Ramp	$\approx {45^{{\circ }}}$	Fingers	5 RH males ($29.3\pm 0.2$ yr)	D	Passive extension of the two fingers in five different equally spaced velocities ranging from ${0.75} \hbox { to } {45}\, {\hbox {rad}/\textrm{s}}$.	[33]
**Healthy and Non-Healthy**^b^						
Repeating Ramp	Not Specified	Shoulder, Elbow, Wrist	3 healthy, (7 Stroke patients)	D	Investigating the effect of strenuous stretching exercise on (individual & cross-coupling) joint stiffness through different velocities passive range of motion (pROM) movements.	[61]
Static	${10^{{\circ }}}$ to ${150^{{\circ }}}$, ${0^{{\circ }}}$ to ${140^{{\circ }}}$, for shoulder and elbow respectively, with increments of ${20^{{\circ }}}$	Shoulder, Elbow	5 healthy adults (25–50 yr), 5 healthy children (13–19 yr), 5 children with disabilities (SMA, MD, Arthrogryposis) (13–18 yr)	D	Measuring the (1) static and (2) maximal isometric push-pull end-point forces and torque in the sagittal plane at different shoulder and elbow joint positions.	[13, 42]
Repeating Ramp	Full	Elbow	10 healthy (6 males) ($48.5\pm 15.2$ yr) and 16 Stroke patients (11 males) ($51.6\pm 14.1$ yr)	E	A clinician moved the subject’s forearm through the elbow’s full ROM at three different velocities: (1) slow (${60}\hbox { to } {99^{{\circ }}/\textrm{s}}$), (2) moderate (100 to ${139^{{\circ }}/\textrm{s}})$ and (3) fast $({140} \hbox { to } {180^{{\circ }}/\textrm{s}})$.	[53]
Repeating Ramp	Healthy: ${107.6} \pm {8.7^{{\circ }}}$, Stroke: ${74.2} \pm {21.5^{{\circ }}}$	Elbow	9 healthy (9 males), ($51.4\pm 24.9$ yr) and 12 chronic Stroke patients (10 males),($53.0\pm 8.5$ yr)	E	The therapist passively moved the subject’s elbow joint at different speeds over the subject’s pROM until a mechanical stop or a 3N m torque limit was reached.	[67]
Ramp-and-Hold	0.15 rad	Elbow	7 healthy (2 males) (21–52 yr) and 5 Obstetric brachial plexus lesion patients (1 male) (24–50 yr)	D	FE ramp-and-hold rotations (0.15 rad, 4 rad/s) while subjects applied four different torque levels (including relaxed 0 N m).	[71]
Ramp-and-Hold	Max ROM from max extension to max FLX	Elbow	20 healthy (5 males) ($71.2\pm 7.2$ yr), 24 PD patients (17 males) ($69.8\pm 7.6$ yr)	E	Examiner passively flexed and extended the subjects’ forearm from maximum EXT to maximum FLX and vice versa for 60 s with short rest in-between direction change.	[29]
Repeating Ramp	Healthy (${142.84} \pm {10.51^{{\circ }}}$), PD (${125.6} \pm {14.35^{{\circ }}}$)	Elbow	11 healthy (10 males) (30–68 yr), 41 Rigidity Dominant patients (35 males) (36–75 yr)	D	Constant speed FE pROM elbow joint movement.	[52]
PRBS	0.03 rad	Elbow	14 healthy, 14 Stroke patients	D	Pseudo-random perturbations (PRBS) are applied to the elbow joint at different positions (${45^{{\circ }}}$ FLX: ${15^{{\circ }}}:{75^{{\circ }}}$ EXT) around its neutral position.	[64]
FP	${60}^{\circ }-120^{\circ }$	Elbow	15 healthy, 15 Hemiparetic (Stroke) patients ($57\pm 10.2$ years)	D	The examiner applied sinusoidal stretches of different frequencies of manual stretch to the elbow joint.	[80]
Pendulum Swing Motion	$\approx {50^{{\circ }}}$	Elbow	11 healthy RH ($59.5\pm 11.8$yr), 11 Stroke patients ($57.7\pm 16.1$yr)	D	Modelled the swing motion of the elbow joint from ${130^{{\circ }}}$ (${0^{{\circ }}}$ full extension) until it reached an equilibrium with the subject lying on a bed and relaxed.	[88]
Ramp	${20^{{\circ }}}$ FLX to ${30^{{\circ }}}$ EXT	Wrist	17 healthy (12 males) ($48\pm 10$yr), (17 Stroke patients, 5 Stroke validation group)	D	Investigated the wrist’s stretch reflex through passive movements (FLX to EXT) at two different constant velocities.	[81]
PRBS	$\approx 2^{{\circ }}$	Wrist	14 healthy (4 males) ($62.9\pm 8.5$yr), (14 PD, 4 males) ($62.6\pm 9.1$yr)	D	Applied perturbations to the wrist joint to investigate the intrinsic and reflex contributions.	[89]
Repeating Ramp	$\approx {30^{{\circ }}}$	Wrist	18 healthy ($67.7\pm 10.9$yr), (19 PD patients ($69.1\pm 9.6$yr))	D	Investigated the co-contraction of muscles during passive movements through two different constant velocities.	[78]

$\approx$, is used to denote approximate values.* PRBS* Pseudo-Random Binary Sequence joint displacement,* FP* Frequency Perturbations;* TP* Torque Pulse;* QR* Quick Release,* STD* Static Torque Displacement;* MVC* Maximum Voluntary Contraction;* MCP* Metacarpophalangeal,* FLX* Flexion,* EXT* Extension,* FE* Flexion/Extension,* ABD* Abduction,* ADD* Adduction,* IER* Interna,/External Rotation,* RUD* Radio-Ulnar Deviation,* PS* Pronation/Supination;* MD* Muscular Dystrophy;* SMA* Spinal Muscular Atrophy,* PD* Parkinson’s Disease,* RH* Right-Handed,* LH* Left-Handed

$^{{\textrm{a}}}$The Perturbation column, for Duchenne Muscular Dystrophy subjects lists experiments carried out with either active or passive components. The remaining groups report only the measurement of the passive joint impedance and not additional measurements with an active component

$^{{\textrm{b}}}$Experiments investigating both healthy and non-healthy (excluding BMD and DMD) subjects, are included only for their healthy-related measurements

We first categorised them based on the type of study population, resulting in five studies including people with DMD, 29 with no pathology, and 13 with and without pathology.

A secondary categorisation based on the investigated joint, dividing them between the entire arm, shoulder, elbow, wrist, wrist and fingers combined, and only fingers (1, 8, 20, 15, 2  and 1, respectively).

The third categorisation is based on the imposed movement or perturbation. The number of studies implementing each perturbation type is shown in Table 3, where each study may include more than one perturbation type. Most studies perturbed the joints by a static hold position, applying a constant velocity (ramp type), or through frequency perturbations.

Out of the five studies examining solely DMD subjects, four applied ramp movements [2, 6, 14, 16] and one included both quick release and frequency perturbations experiments [9], with three investigating the full-arm and two the elbow joint. A description of the most frequently imposed movements follows.

#### Static

In five studies they measured the steady-state passive Jimp at static positions of the joint [13, 39–43]. With sufficient static torque measurements of a combination of positions over the ROM of two joints, a surface area can be created to describe the static passive Jimp, just as Ragonesi et al.[13, 42] did for different elbow and shoulder positions.

**Table 3:** Number of studies implementing each perturbation

Perturbation Type	#
Static	5
Ramp	11
Repeating Ramp	9
Ramp-and-hold	8
Frequency Perturbations	5
Quick-Release	1
Step	2
Pendulum Swing Motion	1
Torque Pulses	3
Pseudo-Random Binary Sequence	5
Random Torque Perturbations	1
Static Torque Displacement	1

#### Frequency perturbations

Frequency perturbations are sinusoidal perturbations that are usually applied for system identification techniques and creating a model of the investigated system [9, 32, 44, 45]. However, these techniques are more appropriate for linear systems [46]. Consequently, to avoid any non-linearities, the frequency perturbations are applied in a typically narrow ROM of less than ${5}^{\circ }$ [9, 32, 44, 45]. The studies identified in this review utilised frequencies of 0.4 to 12 Hz [9, 45, 47], and the majority of joint displacements are in the range of 0.005 to 0.1 rad (${0.3}^{\circ }$ to ${5.7}^{\circ }$), except for MacKay et al. [47], who also investigated 0.5 rad ($\approx {29}^{\circ }$). The only study that implemented frequency perturbation techniques for studying the joint impedance of people with DMD was that of Cornu et al. [9], which applied perturbations of ${3}^{\circ }$ at frequencies of 4 to 12 Hz.

#### Ramp and Variations

Ramp experiments introduce a linear increment of force or position (i.e., constant velocity) to move the joint over a particular ROM. Where constant velocity perturbations allow for the examination of Jimp, linear increment of force perturbations reveal the joint’s compliance (i.e., ‘admittance’). Repeating the same experiment continuously over multiple cycles gives a repeating ramp experiment. As in Lobo-Prat et al. [14], Straathof et al. [6], Lobo-Prat et al. [2], ramp perturbations can be applied on a single joint or on multiple joints simultaneously to obtain the arm’s combined passive Jimp over the workspace.

Another variation, ramp-and-hold, introduces a pause at the end of the movement before switching direction. This pause can be a constant [29, 48–50] or a random value [46, 51]. Different velocities are used in ramp experiments ranging from slow velocities of 0.05 rad/s ($2.86^{{\circ }}/{\textrm{s}}$) [16] up to ${500^{\circ }/{\textrm{s}}}$ [24]. The investigated ROM can vary. For example, the perturbation ROMs for the elbow joint identified are as narrow as ${2^{\circ }}$ [46], wide as $\approx {143^{{\circ }}}$ [52] or even cover the arm’s (horizontal) workspace [2, 6, 16, 53].

Unlike frequency perturbations, the non-linearities over the workspace do not necessarily limit the perturbation’s ROM in ramp studies. However, Klomp et al. [46, 51], Drake and Charles [54] still investigated only a small range of the wrist joint where the stiffness is linear.

Slower velocities (e.g., around $3^{{\circ }}/\textrm{s}$, [16]) can be used to examine the passive Jimp, while higher velocities (e.g., $\ge {60^{{\circ }}/\textrm{s}}$, [53]) can be used to elicit a reflex response affecting the active Jimp [18, 53]. Reflex responses will be further discussed in Subsubsection Reflexes or Volition Dependent Parameters.

As a variation to the rotational ramp-type experiments, Azarsa et al. [55] evaluated the inferior translational admittance of the glenohumeral joint. They evaluated the inferior direction displacement of the shoulder when applying an incremental load of 10 to 80 N in a supine position with ${90^{{\circ }}}$ abduction and external rotation. Also, Gibo et al. [50], reports a linear displacement perturbation (7 mm) of the hand while investigating the contribution of co-contraction of the arm muscles to the Jimp.

### Properties of joint impedance and parameters affecting it

This review has identified several parameters affecting Jimp that should be considered when designing experiments. We divided them into four categories: (1) fixed or biologically dependent, (2) time- or environmentally-dependent, (3) training-related, and lastly, (4) reflexes or volition-dependent.

#### Fixed or biologically dependent parameters

Some attributes are always present and define the Jimp regardless of the time of day, environmental conditions or the person’s physical condition. During a joint’s movement, velocity and acceleration produce dynamic torque components, which add to the total Jimp [40].

***Positional dependence*** With movement, the length changes in musculotendon structures, joint capsules, ligaments, and connective tissues can create passive moments around a joint [40, 41]. Within the muscles, the cross-bridges have spring-like properties [56], which contribute to the positional dependence (i.e., stiffness) component of the passive Jimp [47, 57].

Like a spring’s equilibrium point, the static passive Jimp is at its minimum near the joint’s neutral position of the joint [48]. Around the neutral position, the passive Jimp is linear [25, 54]. Around the neutral position of the wrist (around ${15 ^{{\circ }}}$, Drake and Charles [54]) and the elbow (around ${30 ^{{\circ }}}$, Wiegner and Watts [25]), the passive Jimp is linear. However, this is not true for the extreme positions [48].

For a ${90 ^{{\circ }}}$ abducted arm, Wiegner and Watts [25] identified this neutral elbow position (equilibrium point) to be in the range of ${73^{{\circ }}}\pm {10^{{\circ }}}$ (${107^{{\circ }}}$ relative to the humerus). When the arm is resting besides the subject’s body and pulled by gravity, Jones et al. [58] identified the equilibrium point in the range of ${10^{{\circ }}}$ to ${20^{{\circ }}}$. Whereas, according to Wiegner and Watts [25], the National Aeronautics and Space Administration [59] report a neutral elbow position in a weightless condition to be ${58^{{\circ }}}\pm {24^{{\circ }}}$. Endo et al. [29] made a distinction in the equilibrium for the flexion (${0^{{\circ }}}$ to ${110^{{\circ }}}$) and extension (${110^{{\circ }}}$ to ${10^{{\circ }}}$) movement direction. They report an equilibrium of ${58.1^{{\circ }}}$ (95% CI: ${55.3^{{\circ }}}$ to ${60.9^{{\circ }}}$) for the flexion perturbation and ${61.1^{{\circ }}}$ (95% CI: ${59.2^{{\circ }}}$ to ${62.9^{{\circ }}}$) for the extension perturbation.

For the glenohumeral joint, the intrinsic mechanical properties are coupled over the different rotational axes of the shoulder (e.g., internal/external rotation and horizontal abduction/adduction) [45]. Therefore, different arm positions can lead to variations in the mechanical stiffness and viscous damping [45].

***Multiarticular effects*** In the human body, the force-producing musculotendon structures can span multiple joints, wherein movement at one joint alters the moments applied to an adjacent joint. Consequently, cross-coupling moments between adjacent joints connected by multiarticular muscles can be investigated when moving one of the joints [40, 60, 61].

One such muscle is the m. Triceps Brachii (TB), spanning over the shoulder and elbow joints and acting as an extensor for both of them [41]. The length and moment arm of m. TB changes with varying shoulder and elbow joint angles [41] affecting the passive shoulder extension moment. Landin and Thompson [41] measured the static extension shoulder moments under different elbow and shoulder position combinations. Their reported results of the interaction between the two joints seem contradicting. However, they do identify that the overall passive tissue around the shoulder joint constituted a large proportion of the maximum shoulder extension moment (60–80 %) [41].

Moreover, the passive Jimp of the wrist joint is affected by the muscles and joint position of the forearm and metacarpophalangeal joints [40]. Park et al. [62] measured a 13% coupled stiffness between wrist flexion/extension (FE) and radio-ulnar deviation and Drake and Charles [54] identified in the literature that the forearm rotation counters the pulling direction of the wrist muscles by 12%. Furthermore, the resting position of the fingers can be altered with changes in the wrist position, which change the length of the muscles in the forearm, actuating the fingers [39]. Due to the small weight of the fingers and the hand, the passive musculotendon stiffness properties dominate over the multi-joint arm dynamics, causing the fingers to move towards the resting position [39].

***Contractures*** Joint contractures are typical in DMD, and their development increases with mobility reduction, which increases with wheelchair reliance [1]. Contractures affect the muscle’s operation ROM [25] and can be painful [63]. Combined with connective tissues, adhesions, and abnormal muscle shortening, the passive Jimp rises and the ROM declines [41, 64]. These alter the resting position and the joint’s passive torque-angle relationship [25]. Consequently, passive Jimp directly indicates the contractures [61]. Shortening of the long finger flexor muscles [65, 66] and surgical treatment or conservative management such as splinting, and stretching to elongate the muscle-tendon complex of the joint are common in DMD [63, 65, 66].

***Velocity*** Passive Jimp values tend to increase with rotational speed (from ${11\hbox { to }16^{{\circ }}/\textrm{s}}$ and ${43 \hbox { to } 67^{{\circ }}/\textrm{s}}$), for a given initial elbow joint position of $({60^{\circ } \hbox { and } 90^{\circ }})$, both with and without muscle contraction [48].

Drake and Charles [54] presented that average velocities lower than ${12^{{\circ }}/\textrm{s}}$ do not affect wrist stiffness, and Wu et al. [67] concluded that for velocities of ${90 \hbox { to } 270^{{\circ }}/\textrm{s}}$, the maximum resistance of the elbow increases. Moreover, high velocities can elicit a reflex response at the movement onset (see Strech Reflex paragraph).

***Hysteresis*** Evaluating the Jimp with continuous movement instead of static poses allows for observing the joint’s viscous behaviour and direction dependence during the loading and unloading phases of the torque-angle curve. The curves between these two phases differ, and the area between them indicates the dissipated energy. This energy dissipation results from dry friction (not velocity-dependent) and viscosity (velocity-dependent) and is typical in soft tissues, such as muscles, tendons, and connective tissue [23, 52]. In literature, this phenomenon is also referred to as the hysteresis loop [68]. The shape of the hysteresis loop can change with different amplitude and velocity perturbations [23]. Perturbing a joint around its neutral position reveals the direction dependence of the FE resistance torques [23].

Unlike conventional approaches, Endo et al. [29] models the passive Jimp not with a hysteresis loop, but uses two distinct linear regressions for the flexion and extension phases of the elbow joint, with the cutoff point at the elbow’s equilibrium. The perturbation was, however, manually performed by a single examiner with a simple measurement instrument. So scepticism towards their results is warranted.

***Inertia - Acceleration*** The limb’s inertia affects the passive Jimp when high accelerations occur during the onset of a joint movement or a sudden switch in direction [52]. The inertia of a limb can vary significantly depending on a person’s height and weight [32]. To exclude the effects of inertia from the passive Jimp measurements, studies like Pisano et al. [24], Sepehri et al. [52]  have trimmed the data around the areas of increased acceleration.

#### Time or environmentally dependent parameters

Some parameters vary throughout the day due to environmental changes, the subject’s condition, or muscle state.

***Thixotropy and Temperature*** A skeletal muscle-fibre biological trait, called thixotropy, changes the muscle stiffness depending on the length and contraction history of the muscle [69]. Thixotropy develops through muscle contraction with the detachment of cross-bridges [69] and once the agitated cross-bridge is released it requires time to be re-established. This process transforms the movement from a gel-like to a soluble-like form [70]. A joint’s movement is more prone to the thixotropic effect when lower frequency (e.g.,1 to 3.5 Hz) perturbations are applied to it [70]. Even a 2 s pause still results in an increased stiffness when perturbed in this frequency range [70]. Anguelova et al. [71] incorporated a 15 s rest period between their fast stretch ramp-and-hold protocol to prevent thixotropic force reductions.

Thixotropy is also affected by temperature. The bonding of cross-bridges is greater in cold conditions, as it reduces the muscle’s relaxation rate and diminishes cross-bridge release. In contrast, the cross-bridges release more easily in warmer temperatures [70].

***Short-Range Stiffness*** Short-Range Stiffness (SRS) is a biomechanical property of the musculotendon complex that describes the higher initial stiffness at the beginning of a brief low-amplitude stretch or perturbation. It can be used as a clinical outcome measure of co-contraction and muscle weakness levels [71]. The SRS is attributed to the cross-bridges not detaching quickly enough during rapid movements. It enhances the stability and control of joints during quick transient movements or unexpected forces. The stiffness response is a time-varying parameter since it occurs at an initial perturbation after a 1% change in the length of a muscle after a resting period of about 15 s [54]. In the wrist, this translates to the first ${2^{{\circ }}}$ to ${4^{{\circ }}}$ of the movement [54].

The elastic limit of SRS increases with the perturbation velocity but disappears after approximately ${30}\, {{\textrm{ms}}}$. Once the elastic limit is reached, the stiffness returns to its ‘normal’ resting levels where, assuming a linear system, the muscle behaves like a viscous damper [49]. However, at slower velocities, the SRS is not consistently observed [49].

MacKay et al. [47], observed SRS in the elbow joint with two frequency perturbations (natural frequency in the range of 2 to 3 Hz) and static displacements. During static displacements, SRS stiffness reached values of 4 to 5 N m/rad (four times the stiffness of greater displacements), while during oscillations, SRS reached 14 to 18 N m/rad at the initial 0.1 rad $({5.7^{{\circ }}})$ of the perturbation. When SRS is not of interest, the initial response to the perturbation can be excluded from the analysis. For instance, Pando et al. [72] neglected the first ${2^{{\circ }}}$ of the wrist movement from their analysis.

***Fatigue*** In fatigability, a distinction is made between central (e.g., changes in motor neurons) and peripheral fatigue (e.g., changes at the muscle level). However, these two are interdependent [73]. When fatigued, more motor units are recruited to match the pre-fatigue torque levels. Additionally, an individual’s homeostasis and psychological state influence perceived fatigability [73]. Early onset of fatigue is typical in muscle dystrophies [73, 74] and can affect both Jimp and muscle strength.

Zhang and Rymer [44] observed a reduction in producible torque after investigating the effects of fatigue on the elbow joint in five healthy individuals. They found reduced stiffness and increased viscosity in the elbow as participants actively resisted the perturbations at various torque levels [44]. Similarly, Albanese et al. [73] found that wrist stiffness, modelled using a linear second-order mass-spring-damper system, decreased after fatigue. Here, the participants were asked to naturally grasp the wrist perturbation handle and not intervene or resist the perturbations. These effects are presumably restored within 60 to 90 min [73, 75]. To avoid fatigue during experiments, rest periods can be incorporated between measurements. For instance, Pisano et al. [24] used 10 s rest intervals, whereas  Zhang and Rymer [44] avoided acute fatigue with 10 min breaks.

In contrast to fatigue-induced decreases in Jimp levels, Jones et al. [58] found that the passive muscles were mechanically stiffer, and the resting joint position shifted to a more flexed angle of ${6}^{\circ }$ to $20^{{\circ }}$ following damaging exercise with repetitive eccentric MVC elbow contractions. These damaging effects were observed as early as the next day and lasted up to a week. Bottas et al. [76] also conducted a similar study on exercise-induced muscle damage and found increased passive Jimp and reduced elbow ROM lasting more than eight days.

#### Training related

Engaging in physical activities frequently alters muscle anatomy and can lead to changes in passive Jimp. For instance, Wiegner and Watts [25] found that the upper arm volume significantly correlates with elbow Jimp [25]. Moreover, compared to the non-dominant hand, the dominant hand shows greater stiffness [57]. This could result from changes in the myofibril structure, leading to a higher percentage of slow-twitch fibres in the dominant arm [57]. A higher cross-sectional muscle area is associated with power activities.

On the contrary, stretching exercises can reduce Jimp. A half-hour strenuous stretching session of the shoulder, elbow and wrist joints resulted in a reduced elbow coupling torque in stroke subjects [61]. Additionally, stretching can have a short-term positive effect on the Jimp and both active and passive ROM in chronic stroke patients [77]. This can also be, however, the result of increased tolerance to stretching [77].

#### Reflexes or Volition Dependent Parameters

This category lists parameters which depend on an active response from reflexes or voluntary contraction.

***Co-contraction*** In addition to passive Jimp, agonist–antagonist muscle activation can substantially increase the overall Jimp, improving the stability of a joint [28, 50, 54, 78, 79], either voluntary or as a result of a reflex response. The increase in active Jimp components can vary depending on the joint’s position [78], and there is a trade-off between the gained stability and the metabolic efficiency, as co-contraction is energetically costly [50].

Moreover, anticipation can play a role in the active Jimp. Holmes et al. [79] compared the wrist FE torque between an anticipated and unknown perturbation. They discovered that an anticipated perturbation can lead to an increased Jimp.

***Stretch Reflex*** Several studies have investigated the reflex response to high-velocity perturbations [24, 47, 51, 56, 67, 76, 80, 81]. The reflex loop differentiates reflex contributions from active Jimp by introducing a delay [44]. MacKay et al. [47] identified the onset of this response at approximately 90 ms after the perturbation onset. This response increases the number of active cross-bridges, thereby increasing the active Jimp [56]. At slow velocities, the reflex response is minimal or absent [54, 61, 67, 81]. According to Pisano et al. [24], Wiegner and Watts [25], Wu et al. [67], no reflex response is observed for velocities lower than ${10,\,20\hbox { and }30^{{\circ }}/\textrm{s}}$, respectively.

Several studies [24, 51, 67, 73, 76, 80, 82] used high velocities (${60,\, 100,\,171,\,180,\,230,\,270,\,280\hbox { and }500^{{\circ }}/\textrm{s}}$, respectively) to induce a reflex response. In some participants, Pisano et al. [24] identified the stretch reflex threshold ranging between 60 and ${500}{^{{\circ }}/\textrm{s}}$, whereas in others, there was no reflex response observed at all. Wiegner and Watts [25] report a reflex threshold of ${100^{{\circ }}/\textrm{s}}$ and since the reflex occurs less frequently when the subjects relax more, they discovered that the reflex response is also a function of the applied torque and relaxation state of the subject.

Additionally, the response amplitude varies between muscles and joint positions [82] and ischemia can completely block the reflex response [56].

## Discussion

This study highlights the importance of accurately identifying passive Jimp in individuals with DMD. Unfortunately, in DMD, very little is known about the elevated and non-linear nature of the passive Jimp over the variable passive ROM. Accurate models of Jimp behaviour can help differentiate between voluntary movement intentions, the effects of gravity, and passive Jimp in the UE. This differentiation is crucial for developing force-based control in active assistive arm supports. This review clarifies the terminology around the passive Jimp and provides an overview of ways to identify the passive Jimp in the UE. Additionally, it discusses the parameters that can affect passive Jimp and should be taken into consideration.

The literature reveals considerable variations in the definitions and methodologies used to measure passive Jimp across different studies. Despite differences in terminology, there is a consensus that passive Jimp refers to the resistance against an applied joint rotation caused by the passive biomechanical properties of the muscles, tendons, and tissues around the joint, as well as the limb’s inertia [30]. Given an applied perturbation, the torque-angle relationship reveals the characteristics of the passive Jimp.

This study reviews the various perturbation methods used in the literature to impose a passive movement and measure the applied joint torque. Static, frequency, and ramp-type are the most frequently used movement perturbations for quantifying passive Jimp. The choice between static and dynamic methodologies is at the researcher’s discretion, depending on their specific field of interest. A limitation of this study is its narrative focus on individuals with DMD and the healthy population, which may have restricted the range of identified experimental methods.

Static experiments yield a measure of passive Jimp before movement onset, excluding the velocity effects. Velocity can affect the passive Jimp, with no, to little increase when the movement is slow [54], and higher increase at higher velocities [67]. With frequency perturbations, fast movements over small ROM are provided. Frequency perturbations allow for system identification techniques to model the biomechanical behaviour of a human joint [9, 32, 44]. However, since they only consider a small ROM and assume linear biomechanical behaviour within the considered ROM [46], they do not include the non-linearities over the entire ROM.

When performing the perturbations, the ‘fixed or biologically dependent parameters’ mentioned, such as joint position, velocity, and movement direction, should be considered. The movement direction affects the torque-angle relationship showing a hysteresis loop [23]. The position and velocity parameters can affect the passive Jimp over the entire ROM. Due to the anatomy of the UE muscles, the multiarticular muscles cause cross-coupling torques between the joints, causing the ‘position’ to be a multi-dimensional parameter. Velocities higher than ${60^{{\circ }}/\textrm{s}}$ [24] and ${100^{{\circ }}/\textrm{s}}$ [25] could trigger a reflex response [24, 25, 67]. The reflex response should be avoided when examining only the ‘passive’ Jimp components. For instance, Wiegner and Watts [25] selected a maximal velocity of ${20^{{\circ }}/\textrm{s}}$ to ensure that reflexes would not confound. When perturbations are applied with changing velocities, accelerations and the limb’s inertia also play a role. Understanding the relation of the position and velocity perturbations to the passive Jimp, could provide a basic model of the passive Jimp behaviour.

Additional effects of other parameters, such as the SRS that only affects the onset of a movement [71], the environmental temperature that affects the passive Jimp in cold conditions, or the muscle’s fatigue, could be considered. Exploring the effect of one parameter while eliminating the effects of others may be impossible. However, careful consideration can minimise their impact on passive Jimp. Understanding these parameters and their effects on passive Jimp is essential when designing experiments and interpreting results.

For DMD in particular, most studies have utilised ramp-type perturbations, which involve applying a slow, constant velocity across a large ROM of the joint. These perturbation methods reveal the limited passive ROM as a result of joint contractures [41, 64] and show the non-linear behaviour of the Jimp. Considering the relatively slow arm movements in this application of motorised arm supports [83], the position and velocity dependencies seem more relevant than the inertia dependency. So, ramp-type methods emerge as the most suitable technique for characterising joint impedance over the full ROM and account for the non-linear characteristics of passive Jimp in DMD.

Standardising the terminology around Jimp and methodologies used to identify it, including prioritising the affecting parameters, may lead to better assistive devices. Ultimately improving the quality of life for individuals with DMD.

## Conclusion

In conclusion, to identify the elevated and non-linear behaviour of the passive Jimp in DMD, the ramp-type perturbations, where a constant velocity movement is applied to a larger ROM of the joint, seem most appropriate for the application of accurate and personalised passive Jimp compensation in active assistive arm supports. The identification of passive Jimp does, however, require careful consideration of the parameters affecting it. It is recommended to first examine the influence of the joint’s position, including the influence of multiarticular muscles and the effect of different movement velocities over other parameters; to form the basis of an accurate and personalised Jimp compensation model.

## Data Availability

Not applicable.

## Appendix I: Search Terms

### PuBMed Search Terms

(((stiffness[Title/Abstract]) OR (stiff[Title/Abstract]) OR (stiffnesses[Title/Abstract])) OR ((elasticity[Title/Abstract]) OR (elastance[Title/Abstract]) OR (elastances [Title/Abstract]) OR (elastic[Title/Abstract]) OR (elastical [Title/Abstract]) OR (elastically[Title/Abstract]) OR (elasticities [Title/Abstract]) OR (elastics[Title/Abstract])OR (elasticity[MeSH Terms])) OR ((coactivation[Title/Abstract]) OR (co-activation[Title/Abstract])) OR (contractures[Title/Abstract]) OR (((range[Title/Abstract]) AND (motion[Title/Abstract])) OR (articular range of motion[Title/Abstract]) OR (range of motion[Title/Abstract]) OR (range of motion, articular[MeSH Terms])) OR ((excursion[Title/Abstract]) OR (excursions[Title/Abstract])) OR ((fibrosis[Title/Abstract]) OR (fibrosis[MeSH Terms]) OR (fibro-adipose[Title/Abstract]) OR (fibrotic[Title/Abstract]) OR (fibrosi[Title/Abstract]) OR (fibroses[Title/Abstract]) OR (fibrose[Title/Abstract]))) AND ((muscle[MeSH Terms]) OR (joint[MeSH Terms]) OR (muscle[Title/Abstract]) OR (muscles[Title/Abstract]) OR (muscle’s[Title/Abstract]) OR (joint[Title/Abstract]) OR (joints[Title/Abstract]) OR ( joint’s[Title/Abstract])) AND ((becker[Title/Abstract]) OR (neuromuscular disease[Title/Abstract]) OR (neuromuscular diseases[Title/Abstract]) OR (muscular dystrophy[Title/Abstract]) OR ( muscular dystrophies[Title/Abstract]) OR (muscular dystrophies [MeSH Terms]) OR (duchenne[Title/Abstract]) OR (duchennes[Title/Abstract] ) OR (duchenne’s[Title/Abstract]) OR (muscular dystrophy, duchenne[MeSH Terms])) AND (((upper[Title/Abstract]) AND ((limb[Title/Abstract]) OR (limbs[Title/Abstract]) OR (extremity[Title/Abstract]) OR (extremities[Title/Abstract]))) OR (upper extremity[MeSH Terms]) OR (arm[Title/Abstract]) OR (arms[Title/Abstract]) OR (elbow[Title/Abstract]) OR (elbows[Title/Abstract]) OR (elbow joint[MeSH Terms]) OR (wrist[Title/Abstract]) OR (wrists[Title/Abstract]) OR (wrist joint[MeSH Terms]) OR (shoulder[Title/Abstract]) OR (shoulders[Title/Abstract]) OR (shoulder joint[MeSH Terms])) AND ((adolescent[Filter]) OR (adult[Filter]) OR (youngadult[Filter]))

### SCOPUS Search Terms

( TITLE-ABS-KEY (human) OR INDEXTERMS (human) OR KEY(“normal human”) OR TITLE-ABS-KEY ( Duchenne ) OR INDEXTERMS ( Duchenne ) OR TITLE-ABS-KEY ( duchenne ) OR KEY(DMD) OR KEY(“Duchenne muscular dystrophy”) OR KEY(“muscular dystrophy, duchenne”) OR TITLE-ABS-KEY ( becker ) OR KEY(“becker muscular dystrophy”) OR TITLE- ABS ( “muscular dystrophy” ) OR TITLE-ABS-KEY ( “muscular dystrophies” ) OR INDEXTERMS ( “muscular dystrophy” ) OR TITLE-ABS-KEY ( “neuromuscular disease” ) OR TITLE-ABS-KEY ( “neuromuscular diseases” ) OR KEY(male) OR KEY(biomechanics) ) AND ( TITLE-ABS-KEY ( stiffness ) OR TITLE-ABS ( stiffnesses) OR TITLE-ABS ( stiff ) OR TITLE-ABS ( elasticity ) OR TITLE-ABS ( elasticities ) OR TITLE-ABS ( elastance ) OR TITLE-ABS ( elastances ) OR TITLE-ABS ( elastic ) OR TITLE-ABS ( elastics ) OR TITLE-ABS ( elastical ) OR TITLE-ABS ( elastically) OR INDEXTERMS ( elasticity ) OR TITLE-ABS ( coactivation ) OR TITLE-ABS ( co-activation ) OR TITLE-ABS ( contractures ) OR TITLE-ABS ( excursion ) OR TITLE-ABS ( excursions ) OR TITLE-ABS ( “range of motion” ) OR TITLE-ABS ( range motion ) OR INDEXTERMS ( “range of motion” ) OR INDEXTERMS ( “articular range of motion” ) OR KEY(“range of motion, articular”) OR TITLE-ABS-KEY ( fibrosis) OR TITLE-ABS ( fibrosi ) OR TITLE-ABS ( fibrotic ) OR TITLE-ABS-KEY ( fibro-adipose ) OR TITLE-ABS ( fibroses ) OR TITLE-ABS ( fibrose ) ) AND ( TITLE-ABS-KEY ( muscle ) OR TITLE-ABS ( muscles ) OR TITLE-ABS-KEY ( joint ) OR TITLE-ABS ( joints ) ) AND ( TITLE-ABS-KEY ( “upper extremity” ) OR TITLE-ABS-KEY ( “upper extremities” ) OR TITLE-ABS-KEY ( arm ) OR TITLE-ABS-KEY ( arms ) OR TITLE-ABS-KEY ( wrist ) OR TITLE-ABS-KEY ( shoulder ) OR TITLE-ABS-KEY ( shoulders ) OR TITLE-ABS-KEY ( elbow ) OR KEY ( “elbow joint” ) OR KEY ( “shoulder joint” ) OR INDEXTERMS ( elbow ) OR KEY ( “wrist joint” ) OR KEY ( wrist ) OR TITLE-ABS-KEY ( “upper limb” ) OR TITLE-ABS-KEY ( “upper limbs” ) )

AND ( SUBJAREA ( neur ) OR SUBJAREA ( engi ) ) AND ( EXCLUDE ( DOCTYPE,“no” ) ) AND ( LIMIT-TO ( LANGUAGE,“English” ) ) AND ( LIMIT-TO ( EXACTKEYWORD,“Adult” ) OR LIMIT-TO ( EXACTKEYWORD,“Young Adult” ) OR LIMIT-TO ( EXACTKEYWORD,“Adolescent” ) OR EXCLUDE ( EXACTKEYWORD,“Diagnostic Imaging” ) OR EXCLUDE ( EXACTKEYWORD,“Aged, 80 And Over” ) OR EXCLUDE ( EXACTKEYWORD,“Leg” ) OR EXCLUDE ( EXACTKEYWORD,“Knee” ) OR EXCLUDE ( EXACTKEYWORD,“Trunk” ) OR EXCLUDE ( EXACTKEYWORD,“Stroke Rehabilitation” ) )

### IEEEXplore Search Terms

(((stiffness) OR (stiff) OR (stiffnesses)) OR ((elasticity) OR (elastance) OR (elastances) OR (elastic) OR (elastical) OR (elastically) OR (elasticities) OR (elastics)) OR ((coactivation) OR (“co-activation”)) OR (contractures) OR (((range) AND (motion)) OR (“articular range of motion”) OR (“range of motion”)) OR ((excursion) OR (excursions)) OR ((fibrosis) OR (“fibro-adipose) OR (fibrotic) OR (fibrosi) OR (fibroses) OR (fibrose))) AND ( (muscle) OR (muscles) OR (joint) OR (joints) ) AND ((becker) OR (neuromuscular disease) OR (neuromuscular diseases) OR (muscular dystrophy) OR (muscular dystrophies) OR (duchenne) OR (duchennes) OR (duchenne’s)) AND (((upper) AND ((limb) OR (limbs) OR (extremity) OR (extremities))) OR (“upper extremity”) OR (arm) OR (arms) OR (elbow) OR (elbows) OR (“elbow joint”) OR (wrist) OR (wrists) OR (“wrist joint”) OR (“shoulder joint”))

### WebOfScience Search Terms

(ALL=(((stiffness) OR (stiff) OR (stiffnesses)) OR ((elasticity) OR (elastance) OR (elastances) OR (elastic) OR (elastical) OR (elastically) OR (elasticities) OR (elastics)) OR ((coactivation) OR (co-activation)) OR (contractures) OR (((range) AND (motion)) OR (articular range of motion) OR (range of motion)) OR ((excursion) OR (excursions)) OR ((fibrosis) OR (fibro-adipose) OR (fibrotic) OR (fibrosi) OR (fibroses) OR (fibrose)))) AND (ALL=((muscle) OR (joint) OR (muscles) OR (muscle’s) OR (joints) OR (joint’s))) AND (ALL=((becker) OR (neuromuscular disease) OR (neuromuscular diseases) OR (muscular dystrophy) OR (muscular dystrophies) OR (duchenne) OR (duchennes) OR (duchenne’s))) AND (ALL=(((upper) AND ((limb) OR (limbs) OR (extremity) OR (extremities)) OR (arm) OR (arms) OR (elbow) OR (elbows) OR (elbow joint) OR (wrist) OR (wrists) OR (wrist joint) OR (shoulder joint))))

## Abbreviations

DMD: Duchenne muscular dystrophy
UE: Upper extremity
ROM: Range of motion
Jimp: Joint impedance
MeSH: Medical subject headings
TB: Triceps brachii
FE: Flexion/extension
SRS: Short-range stiffness
